# Spinal-pelvic sagittal parameters in patients with gluteal muscle contracture: an imaging study

**DOI:** 10.7717/peerj.13093

**Published:** 2022-03-15

**Authors:** Jiyi Liu, Pengzhou Huang, Guanwei Jiang, Liang Gao, Mengdi Zhang, Xueping Dong, Wentao Zhang, Xintao Zhang

**Affiliations:** 1 Shantou University, Shantou, China; 2 Peking University Shenzhen Hospital, Shenzhen, China; 3 Center of Experimental Orthopaedics, Saarland University Medical Center, Homburg/saar, Germany; 4 Sino Euro Orthopaedics Network (SEON), Berlin, Germany

**Keywords:** Gluteal muscle contracture, Spine, Pelvis, Spinal-pelvic, Imaging

## Abstract

**Background:**

Gluteal muscle contracture (GMC) may cause abnormal spinal alignment as well as hip and pelvic deformities. The spine-pelvis alignment of GMC patients is unclear. This study aimed to describe the spine-pelvis sagittal alignment in patients with GMC and to explore the impact of GMC on the pathogenesis of low back pain (LBP).

**Methods:**

Radiological analysis was performed in 100 patients with GMC and 100 asymptomatic volunteers who acted as the control group. Sagittal parameters were measured by two independent raters and their averages were presented on lateral radiographs of the whole spine, including pelvic incidence (PI), sagittal vertical axis (SVA), pelvic tilt (PT), lumbar lordosis (LL), sacral slope (SS), thoracic kyphosis (TK), and the relationship between PI and LL (expressed as PI-LL). All cases were categorized into one of three classes based on the apex position of lumbar lordosis and were further divided into three groups by the PI value. The GMC and control parameters were compared and the correlations between the parameters in the GMC group were analysed.

**Results:**

The PI value of the GMC group was significantly less than that of the control group (42.38 ± 10.90° *vs* 45.68 ± 7.49°, *P* < 0.05). There was no difference found between the key parameters (SVA, PT, and PI-LL), which correlated with outcomes in adult deformity. No differences of SS were found between the two groups (*P* > 0.05). The GMC group showed lower average LL (42.77 ± 10.97° *vs* 46.41 ± 9.07°) and TK (17.34 ± 9.50° *vs* 20.45 ± 8.02°) compared with the control group (*P* < 0.05). LL was correlated with PI, SS, PT, TK (*P* < 0.01) and SVA (*P* < 0.05). TK and SVA were not correlated with any parameters except LL and pairwise correlations were found among PI, SS, and PT. There were no differences found between the distributions of the lumbar lordosis apex of GMC and the control but the range of SS in apex groups 3 and 4 did differ. GMC patients had the most small-PI value (44%) while approximately 64% of asymptomatic individuals had a normal PI. Interobserver variability was sufficient for all parameters calculated by the intraclass correlation coefficient (ICC).

**Conclusions:**

Gluteal muscle contracture causes a low PI which may contribute to low back pain. Patients with GMC present the same global sagittal spinal-pelvic balance as asymptomatic individuals due to a compensatory mechanism through excessive flat lumbar and thoracic curves. Future studies on the relationship between spinal-pelvic sagittal and coronal alignment and low back pain are needed to understand the mechanical forces involved in the onset of GMC.

## Introduction

Gluteal muscle contracture (GMC) is currently recognized as a clinical syndrome with multiple etiologies. It is characterized by fibrosis and contracture of the gluteal muscles and the surrounding fascia (Zhang et al., 2015; Zhao et al., 2010). The degree of bilateral hip contracture is often unequal, resulting in an uneven force on the pelvis and subsequent pelvic tilt. GMC may affect the morphological development of the pelvis (Sautet et al., 2018). Clinical manifestations include usual abnormalities in the hip joint, including hip snapping, abnormal gait, hip joint movement disorder, and difficulty for patients to keep their knee joints close together during squatting (Zhang et al., 2017). Some patients with GMC presented with spinal symptoms, especially low back pain (LBP). GMC may induce related symptoms by affecting the spine and pelvis, therefore, we studied the relevant research.

Previous studies have shown that spinal-pelvic sagittal alignment is closely related to many spinal diseases (Labelle et al., 2004; Boulay et al., 2006; Vaz et al., 2002) and may be associated with the clinical symptoms and outcomes of these diseases. Recently, PI has been found in lumbar degenerative diseases that may be associated with lower back pain (Chaléat-Valayer et al., 2011; Schwab et al., 2010; Duval-Beaupère, Schmidt & Cosson, 1992). Prior studies have demonstrated that spinal-pelvis sagittal alignment was an independent predictor of pain and disability (Schwab et al., 2010; Glassman et al., 2005; Rose et al., 2009). Sagittal vertical axis (SVA), pelvic tilt (PT), and pelvic incidence-lumbar lordosis mismatch (PI-LL) were confirmed to be three main sagittal parameters that correlated with outcomes in adult deformity. SVA is a parameter of the efficiency degree of the overall upright position (Rose et al., 2009). PT was used to measure the retroversion of the pelvis in a standing position (Vialle et al., 2005). An increase in PT leads to an increase in compensatory muscle effort to stabilize the pelvis and keep the body upright. PT was positively correlated with worsening pain (Lafage et al., 2009). More balanced spines exhibit a smaller PT. Asymptomatic individuals presented a substantial correlation between pelvic incidence (PI) and lumbar lordosis (LL) (Vialle et al., 2005). Compared with PI, patients with too few degrees of LL showed poor clinical outcomes in pain and disability (Rose et al., 2009). Patients with chronic low back pain were also statistically more likely to have a vertically oriented sacrum (less sacral slope (SS)) and less LL (Evcik & Yücel, 2003).

However, the spinal-pelvis alignment of GMC patients is not sufficiently clear and the sagittal features of the spinal-pelvis relationship in patients with GMC have not been reported. Therefore, this study aimed to describe the characteristics of the sagittal alignment in patients with GMC and to explore the impact of GMC on the pathogenesis of LBP.

## Materials and Methods

The single-center, single-blind, prospective study was approved by the Regional Ethics Committee of Peking University, Shenzhen Hospital (Reference: (research) (2017) 008^th^). Each participant provided their written consent with a signature for confirmation (Gao et al., 2020).

A sample of 100 patients was needed to achieve a 95.77% power to reject the null hypothesis of equal means when the population mean difference was 0.3–(−1.1) = 1.4 mm of the general spine with standard deviations of 3.0 for the degeneration group and 2.3 for the normal group and a significance level (alpha) of 0.05 using a two-sided, two-sample, unequal-variance t-test, based on a previous report (Yang et al., 2014).

### Inclusion criteria

Patients with GMC were randomly enrolled in this study from January 2019 to October 2020. The clinical and imaging findings met the conditions for the diagnosis of GMC. An equal number of asymptomatic volunteers, whose age and gender matched the GMC group, were included as the control group. Anteroposterior and lateral roentgenogram of the entire spine and pelvis were taken for all of the subjects. Each participant was asked to stand in an upright and comfortable posture with hands placed on an armrest.

### Exclusion criteria

Objects with any of the following conditions were excluded:

1. The subjects in the control group with LBP, leg pain, lumbar spondylolisthesis, scoliosis, and other spinal disorders were excluded through history taking and imaging examination
2. Patients with histories of spinal and pelvic operation, trauma, and lumbar spondylolisthesis were excluded.

A total of 100 patients (35 males and 65 females), aged 17–46 (28.50 ± 5.41) years old were enrolled in the GMC group. Of these, there were 16 patients for whom we had the Visual Analogue Scale (VAS) adopted for LBP. A total of 100 asymptomatic individuals (35 males and 65 females), aged 17–47 (28.56 ± 7.22) years old were included as the control group. The volunteers were mainly selected from medical staff, medical students, and nursing personnel affiliated with the hospital where the study was performed.

### Measures

**Figure 1:**
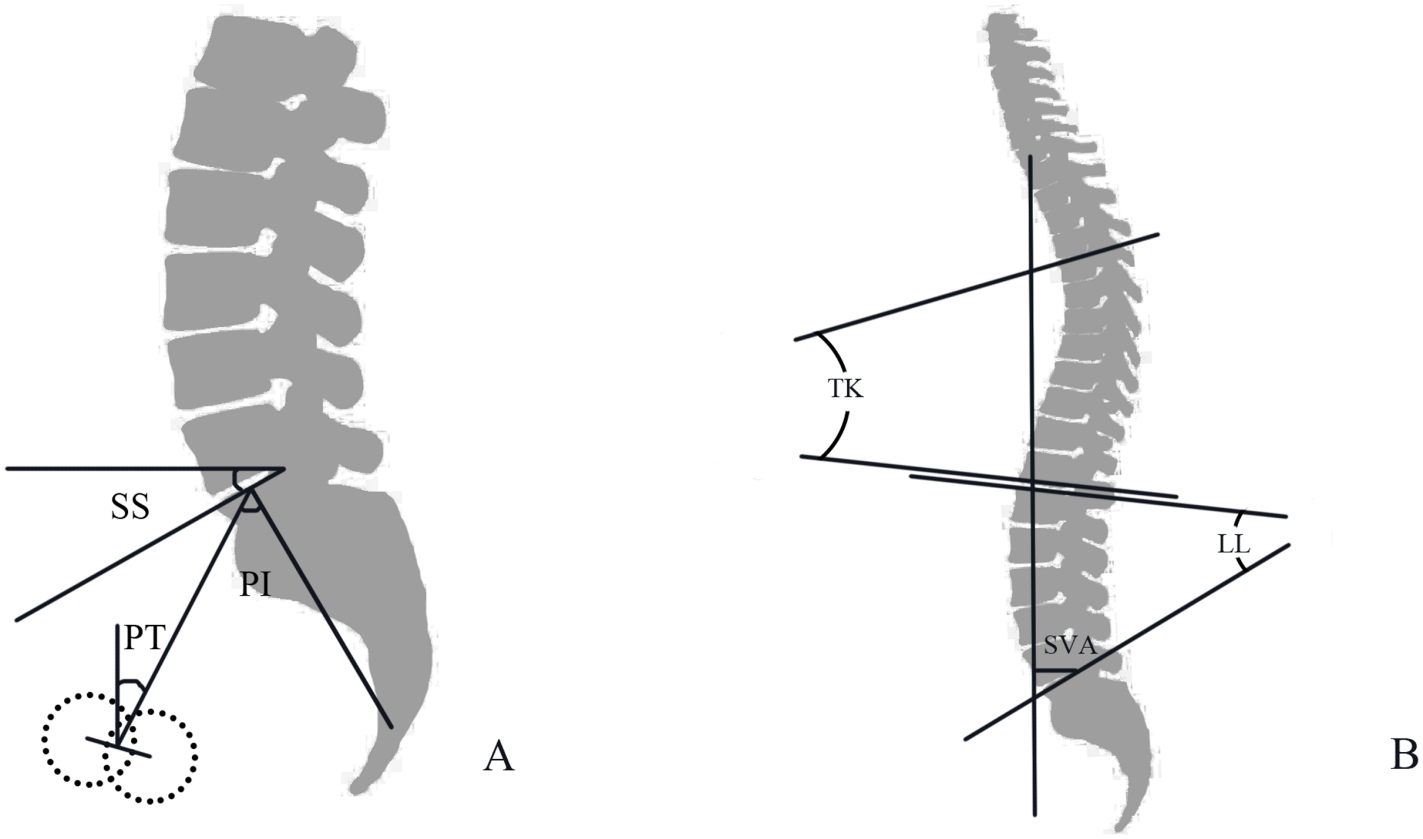
(A) Diagrammatic sketch of PI, Pelvic Incidence angle; PT, pelvic tilt; SS, sacral slope; (B) Diagrammatic sketch of SVA, sagittal vertical axis; TK, thoracic kyphosis; LL, lumbar lordosis.

Imaging standards: All patients and asymptomatic individuals underwent full-length spine radiography using the hospital’s GE646DR photography system (General Electric Co., Boston, USA). An image of the full-length spine (including both hip joints) in the weight-bearing position was used and the image was automatically stitched together after continuous exposure. Imaging measurement technology was used to record the deformity according to the standardized manual (Roussouly et al., 2005). Picture archiving and communication systems (PACS) were used to measure the distance (SVA), and SURGIMAP software (Surgimap, Methuen, MA, USA) was used to measure the angle parameters (Fig. 1).

Spinal sagittal parameters: sagittal vertical axis (SVA), the horizontal distance between the plumb line of the seventh cervical vertebra and the posterior superior angle of the sacral endplate; Cobb angle of thoracic kyphosis (TK) of T5 to T12 in the sagittal plane; lumbar lordosis (LL) angle, the angle between the upper endplate and S1 upper endplate.

Pelvic sagittal parameters: pelvic incidence (PI), the midpoint of the endplate and the centreline of both femoral heads make a straight line, and the angle between the straight line and the vertical line of the S1 upper endplate; pelvic tilt (PT), the angle between the straight line (the midpoint of the endplate and the center line of both femoral heads) and the plumb line; sacral slope (SS), the angle between the tangent line of the endplate and the horizontal line on S1.

All the stitched images were analyzed offline by two independent observers. Relevant parameters were also analyzed twice by the two raters and their averages were presented.

### Grouping

All subjects were divided into three groups according to the PI value. Subjects were also grouped by the position of the apex based on the classification from Roussouly et al. (2005):

1. The cases were grouped by the different values of PI. According to prior studies (Zhu et al., 2014; Fei et al., 2017), a normal PI value was approximately 45°. Three groups were established: namely low PI (PI *≤* 40°), middle PI (40° ≤ PI < 50°), and high PI (PI ≥ 50°).
2. The sagittal alignment of the spinal-pelvic was classified into three types according to the position of LL apex: I (lumbar 3 vertebral, L3), II (lumbar 4 vertebral, L4), III (lumbar 5 vertebral, L5).
3. All subjects were divided into three groups according to the value of SS in different apex groups, as mentioned above.

### Statistical analysis

All data were analyzed using SPSS 26.0 software (IBM Corp., Amonk, NY, USA). However, the sample-size calculation were performed using PASS 15.0. The continuous variables were summarized by mean ± standard deviation (SD) while categorical variables were summarized by number and percentage. The statistical level of significance was set at 0.05. An Independent-sample Student’s *t*-test or Mann-Whitney U-test was used to compare the parameters, especially PI, between the GMC group and control group; the categorical variable was described as rate (100 percent). We used a Pearson correlation to analyse the correlation between parameters in the GMC group. A χ2 -test was used to analyze the frequency components of each group. The intraobserver reproducibility was evaluated by means of intraclass correlation coefficient (ICC).

## Results

### General data

The average age of GMC patients, which included 35 males and 65 females, was 28.5 ± 5.4 years (range: 17–46 years). The control group also included 35 males and 65 females with a mean age of 28.6 ± 7.2 which ranged from 17 to 47. The VAS of the 16 cases with data was 3.13 ± 0.34 for LBP. No significant difference was found for age and gender distribution using the independent sample *t*-test and χ2 -test between the GMC group and the control group (P > 0.05), indicating that age and gender were well-matched.

### Intraobserver variability

The results of the intraobserver agreement were excellent. ICC was 0.997, 0.985, 0.966, 0.965, 0.988, and 0.968 for SVA, TK, LL, PI, SS, and PT (*P* < 0.01), respectively.

### Radiographic parameters

The measurement and comparison results of the radiographic parameters are shown in Table 1. It should be noted that the data of SVA and PI-LL measured in this study did not conform to normal distribution. Therefore, SVA and PI-LL were represented by the median (interquartile range), and the Mann-Whitney U test was used to compare the GMC group and the control group. The correlation among the parameters in the GMC group is shown in Table 2. The independent sample *t*-test indicated that there was difference in PI between these two groups (42.38 ± 10.90° *vs* 45.68 ± 7.49°, *P* < 0.05). The LL (42.77 ± 10.97° *vs* 46.41 ± 9.07°, *P* < 0.05) and TK (17.34 ± 9.50° *vs* 20.45 ± 8.02°, *P* < 0.05) of GMC group were lower, while its SVA (9.65 (−20.34 to 27.35) *vs* 0.03 (−21.06 to 13.45), *P* > 0.05), PT (8.73 ± 8.92° *vs* 10.92 ± 6.95°, *P* > 0.05), SS (33.56 ± 8.76° *vs* 34.79 ± 6.48°, *P >* 0.05) and PI-LL (−1.45 (−7.70 to 6.55) *vs* −1.98 (−7.08 to 5.74), *P* > 0.05) did not reach statistical significance compared to the control group. Previously, LL and SVA were shown to be related to a surgical effect (Schwab et al., 2013; Tsai et al., 2011). LL was significantly correlated with PI, SS, PT, TK (*P* < 0.01). However, SVA did not show significant difference with PI, SS, PT, TK (*P* > 0.05) except for LL (*P* < 0.05).

**Table 1:** Parameter measurement results.

Group	SVA (mm)**	TK (°)*	LL (°)*	PI (°)*	SS (°)	PT (°)	PI-LL
GMC (100)	9.65 (−20.34 to 27.35)	17.34 ± 9.05	42.77 ± 10.97	42.38 ± 10.90	33.56 ± 8.76	8.73 ± 8.92	−1.45 (−7.70 to 6.55)
Control (100)	0.03 (−21.06 to 13.45)	20.45 ± 8.02	46.41 ± 9.07	45.68 ± 7.49	34.79 ± 6.48	10.92 ± 6.95	−1.98 (−7.08 to 5.74)

**Notes:**

SVA, sagittal vertical axis; TK, thoracickyphosis; Ll, lumbarlordosis; PI, pelvic incidenceangle; PT, pelvic tilt; SS, sacral slope; PI-LL, pelvic incidence lumbar lordosis mismatch.

** SVA was represented by median (interquartile range), and the Mann-Whitney u-test was used for comparison between the GMC group and the control group.

* *P* < 0.05.

**Table 2:** Correlation among parameters in GMC.

	PI	SS	PT	TK	SVA
LL	0.491**	0.876**	−0.263**	0.343**	−0.224*
PI		0.603**	0.617**	0.162	0.135
SS			−0.253*	0.090	0.028
PT				0.111	0.139
TK					0.028

**Notes:**

SVA, sagittal vertical axis; TK, thoracic kyphosis; LL, lumbar lordosis; Pl, pelvic incidence angle; PT, pelvic tilt; SS, sacral slope.

* *P* < 0.05.

** *P* < 0.01.

The χ2 -test showed that there were fewer normal PI and more low PI in the GMC group, while the control group had the most normal PI and the fewest low PI (*P* < 0.05) for cases grouped by PI (Table 3). High PI occupied the middle position in both patients and asymptomatic subjects. The χ2 -test suggested that there was no difference among the three groups in the distribution of apex (*P* > 0.05) for the position of lordosis apex (Table 4). Nonetheless, the range of SS in L3 (38.42 ± 4.69° *vs* 47.18 ± 0.88°, *P* < 0.01) and L4 (34.04 ± 8.64° *vs* 36.50 ± 5.09°, *P* < 0.05) (Table 5) showed a statistical difference between the GMC group and

**Table 3:** Distribution of pelvic incidence (PI) groups (cases (%)).

	I	II	III
GMC (%)	44 (44)	36 (36)	20 (20)
Control (%)	23 (23)	52 (52)	25 (25)
$\chi^2$		10.047	
*P*		0.007*	

**Notes:**

I, PI < 40°; Il, 40° ≤ PI < 50°; II, Pl ≥ 50°.

* *P* < 0.01.

**Table 4:** Classification of the apex in GMC group and control group (cases (%)).

	APEX		
	L3	L4	L5
GMC (%)	6 (6)	74 (74)	20 (20)
Control (%)	5 (5)	64 (64)	31 (31)
$\chi^2$		3.188	
*P*		0.203	

**Note:**

L3, apex on lumbar 3 vertebral; L4, apex on lumbar 4 vertebral; L5, apexon lumbar 5 vertebral.

**Table 5:** Range of sacral slope(ss) in the different apex of GMC group and control group.

	APEX		
	L3	L4	L5
GMC (*n* = 100)	38.42 ± 4.69	34.04 ± 8.64	30.34 ± 9.39
Control (*n* = 100)	47.18 ± 0.88	36.50 ± 5.09	29.24 ± 4.49
*t*	−4.479	−2.076	0.491
*P*	0.005*	0.040*	0.628

**Notes:**

L3, apex on lumbar3 vertebral; L4, apex on lumbar 4vertebral; L5, apex on lumbar5 vertebral.

* *P* < 0.01.

## Discussion

The sagittal alignment of the spine and pelvis was found to be highly variable in different subjects and the imaging parameters revealed a number of differences between GMC patients and asymptomatic participants.

### Pelvic incidence in patients with gluteal muscle contracture

Pelvic incidence was associated with low back pain (Chaléat-Valayer et al., 2011). The PI was lower in patients with lumbar degeneration, as discovered by Yang et al. (2014) who hypothesized that a low PI would direct more vertical pressure on the disc and cause degeneration. Furthermore, they also assumed that a low PI may reduce the average PI in the control group with asymptomatic degeneration. Moreover, in a study on the spinal-pelvic sagittal alignment in young Chinese patients with lumbar disc herniation (LDH), low PI may have caused degeneration of the disc, which was associated with low back pain (Chaléat-Valayer et al., 2011; Fei et al., 2017). The sagittal shape of the pelvis is relatively stable, and it will not change with age or degeneration in adulthood. Therefore, pelvic incidence can be used as a permanent information source for the original shape of the spinal sagittal plane (Sautet et al., 2018; Lafage et al., 2009; Mac-Thiong, Labelle & Roussouly, 2011; Mac-Thiong et al., 2004). You et al. (2020) found that in female patients with GMC, the pelvis becomes narrower, which affects the normal delivery of infants among women of childbearing age. It should be surmised that the shape of the pelvis may be impacted by GMC during growth, which leads to low PI. We compared 100 GMC patients and 100 asymptomatic volunteers. The results show a significant difference in PI between these two groups. The mean PI of GMC patients was smaller than the mean PI of the control group. In summary, GMC may cause a low PI which contributes to low back pain. However, not all the GMC patients with low PI presented with LBP. This may be due to compensation by stronger muscles, greater endurance, or spinal-pelvic auto-regulation.

### Sagittal alignment in patients with gluteal muscle contracture

Sagittal alignment of the spine has been described as an independent predictor of pain and disability in patients with a spinal deformity (Glassman et al., 2005; Rose et al., 2009; Schwab et al., 2010). Substantial evidence in the earlier literature revealed the correlation of sagittal alignment with HRQoL (Kim et al., 2011; Heo et al., 2015; Kumar, Baklanov & Chopin, 2001). Schwab et al. (2010) established ground-breaking correlations among SVA, PT, and PI-LL, and pain and ODI scores. The threshold of SVA to distinguish between patients’ HRQoL is around 50 mm (Schwab et al., 2010) and this is considered to be the highest degree of efficiency of the global upright trunk position a patient can attain. The more positive the SVA, the greater the patients’ pain and disability and the poorer the function. There is also a positive correlation found in a study on truncal inclination between PT and worsening pain and disability scores (Lafage et al., 2009). An increase of PT causes an increase in the pelvic torque, which is the torsion moment of the body weight and the ground reaction force respectively applied to the pelvis at the sacrum and acetabula. To stabilize the pelvis and keep the body upright there is an increase in the compensatory muscle effort. A substantial correlation between PI and LL is shown in asymptomatic individuals (Vialle et al., 2005). Patients with too few degrees of LL when compared with PI are shown to have poor clinical outcomes for pain and disability.

However, this study showed that SVA, PT, and PI-LL were not different between the GMC group and the control group. The effect of impacting the global sagittal balance of spinal-pelvic was inconspicuous when compared to the asymptomatic volunteers in this study. It was inferred by the results that there may be compensation for this influence. Previous studies have suggested that there appears to be a compensatory mechanism with the loss of the spinal sagittal balance. The pelvis may compensate for sagittal balance by tilting backward, which leads to increasing PT. However, most asymptomatic individuals have a PT less than 21 degrees. In the present study, the mean PT of the GMC group and the control group were not greater than 21 degrees. Barrey et al. (2007) presumed that backward rotation of the pelvis may compensate for the loss of LL and sagittal imbalance since SS is equivalent to the lower lumbar spine which accounts for more than 2/3 of LL. According to the geometric relationship, PI = PT + SS. The pelvis was thought to be involved in compensation when SS < PT. It was shown that the mean SS was greater than PT in both the GMC group and the control group. These results suggested that the compensation mechanism did not include the increase of PT and decrease of SS in the patients with GMC. It is noteworthy that the decrease of TK was involved in the compensatory mechanism in which there was a loss of sagittal balance in young LDH patients, which was indicated by Fei et al. (2017). We also found that the TK of the GMC group was smaller than that of the control group.

GMC did not make an evident impact on the loss of global sagittal balance of the spine. It is also possible that the sagittal imbalance was offset by an inconspicuous compensation, which may be another reason that GMC patients with low PI did not suffer from LBP.

### Lumbar curve and sacral slope in patients with gluteal muscle contracture

**Figure 2:**
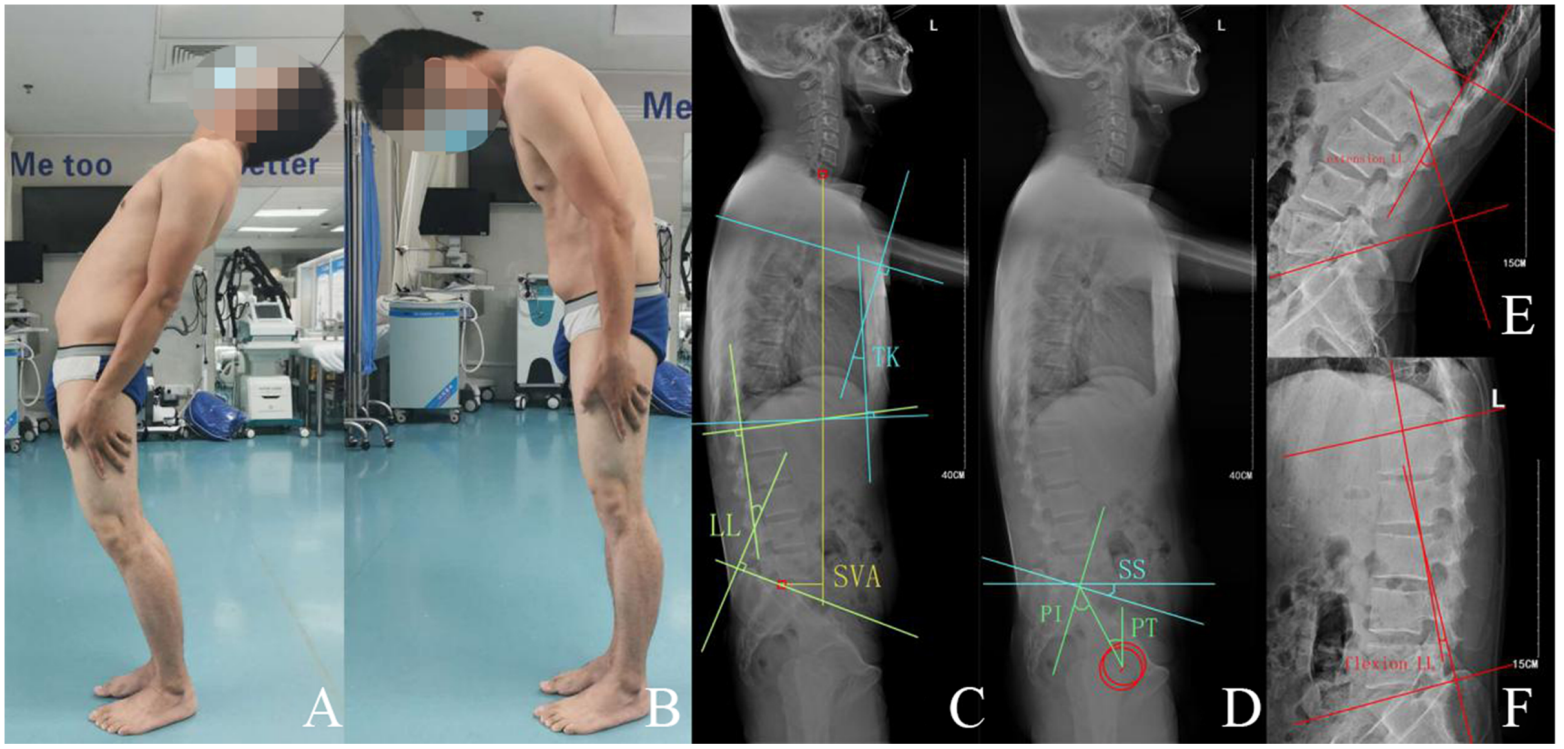
32 years old male with gluteal muscle contracture complained of low back pain and limitation of lumbar flexion. (A, B) Extension and flexion of lumbar, (C, D) standing lateral radiograph of spine-pelvis, SVA 61.6 mm, LL 15.4°, TK 12.8°, PT 21.6°, PI 41.3°, SS 19.7°, (E, F) extension-LL −50.2°, flexion-LL 5.4°.

The correlation between SS and the LL apex was discovered by Roussouly et al. (2005). The different LL apex had a unique SS range since LL is correlated with PI, which contributes to LBP. The relationship between the lumbar curve and SS in patients with GMC was explored in this study through an analysis of the position of the LL apex. There was no difference among the three groups in the distribution of the apex. The least common category (L3) contained six patients (GMC group) *vs* five individuals in the control group, whereas the most common category (L4) contained 74 patients (GMC group) *vs* 64 individuals in the control group. The majority of the inflation points of the lumbar curve in GMC patients were located on the 4^th^ lumbar vertebral body, which was also seen in the control group. However, compared to the control group, the range of SS in patients with GMC was greater when the apex was located on the L5 vertebral body. Remarkably, asymptomatic individuals presented with the same SS range as the control participants in a study by Roussouly et al. (2005). It suggested that the correlation between SS and the LL apex may fluctuate in response to the fibrotic gluteal. Further, according to the apex classification in this study, the range of SS in L3 and L4 showed a statistical difference between the GMC group and the control group, revealing that the mean SS of the GMC group was smaller than that of the control group. We speculated that the lumbar curve may be impacted by the gluteal muscle contracture and found that the lumbar range of motion in patients with GMC was limited, especially in regard to lumbar flexion Fig. 2. Therefore, the LL of the two groups differed and the LL of the GMC group was smaller in comparison to the control group, indicating that the lumbar curve of patients with GMC decreased.

Asymptomatic individuals showed no direct evidence that a gluteal muscle contracture affected the global spinal-pelvic sagittal alignment *vs* the partial spine and pelvis, as indicated by a decrease in the LL and TK in patients with GMC.

### Limitations

This study was limited by a lack of previous research on the correlation between GMC and clinical spinal symptoms. Further, there was a large range of normal individual parameters measured, indicating that there may be large individual differences in the sagittal shape of the spine-pelvis. Future research should seek to improve and clarify the effect of GMC on the shape and alignment of the spinal-pelvic sagittal plane.

## Conclusion

Gluteal muscle contracture causes a low PI which may contribute to low back pain. Patients with GMC present the same global sagittal spinal-pelvic balance as the asymptomatic individuals by compensatory mechanism through the excessive flat lumbar and thoracic curves. To clarify the mechanical forces involved in GMC onset, further investigations between both relations of spinal-pelvic sagittal and coronal alignment and low back pain are needed.

## Supplemental Information

10.7717/peerj.13093/supp-1 Supplemental Information 1 Original data by 2 raters.

## Additional Information and Declarations

### Data Availability

The following information was supplied regarding data availability: The raw data are available in the Supplemental File.
